# Cystic Fibrosis Transmembrane Conductance Regulator (CFTR) in Human Lung Microvascular Endothelial Cells Controls Oxidative Stress, Reactive Oxygen-Mediated Cell Signaling and Inflammatory Responses

**DOI:** 10.3389/fphys.2020.00879

**Published:** 2020-07-29

**Authors:** Maha Khalaf, Toby Scott-Ward, Adam Causer, Zoe Saynor, Anthony Shepherd, Dariusz Górecki, Anthony Lewis, David Laight, Janis Shute

**Affiliations:** ^1^School of Pharmacy and Biomedical Sciences, Institute of Biological and Biomedical Sciences, University of Portsmouth, Portsmouth, United Kingdom; ^2^Department of Sport and Exercise Science, University of Portsmouth, Portsmouth, United Kingdom

**Keywords:** endothelium, CFTR, cystic fibrosis, inflammation, oxidative stress, cell signaling

## Abstract

**Background:**

Perturbation of endothelial function in people with cystic fibrosis (CF) has been reported, which may be associated with endothelial cell expression of the cystic fibrosis transmembrane conductance regulator (CFTR). Previous reports indicate that CFTR activity upregulates endothelial barrier function, endothelial nitric oxide synthase (eNOS) expression and NO release, while limiting interleukin-8 (IL-8) release, in human umbilical vein endothelial cells (HUVECs) in cell culture. In view of reported microvascular dysfunction in people with CF we investigated the role of CFTR expression and activity in the regulation of oxidative stress, cell signaling and inflammation in human lung microvascular endothelial cells (HLMVECs) in cell culture.

**Methods:**

HLMVECs were cultured in the absence and presence of the CFTR inhibitor GlyH-101 and CFTR siRNA. CFTR expression was analyzed using qRT-PCR, immunocytochemistry (IHC) and western blot, and function by membrane potential assay. IL-8 expression was analyzed using qRT-PCR and ELISA. Nrf2 expression, and NF-κB and AP-1 activation were determined using IHC and western blot. The role of the epidermal growth factor receptor (EGFR) in CFTR signaling was investigated using the EGFR tyrosine kinase inhibitor AG1478. Oxidative stress was measured as intracellular ROS and hydrogen peroxide (H_2_O_2_) concentration. VEGF and SOD-2 were measured in culture supernatants by ELISA.

**Results:**

HLMVECs express low levels of CFTR that increase following inhibition of CFTR activity. Inhibition of CFTR, significantly increased intracellular ROS and H_2_O_2_ levels over 30 min and significantly decreased Nrf2 expression by 70% while increasing SOD-2 expression over 24 h. CFTR siRNA significantly increased constitutive expression of IL-8 by HLMVECs. CFTR inhibition activated the AP-1 pathway and increased IL-8 expression, without effect on NF-κB activity. Conversely, TNF-α activated the NF-κB pathway and increased IL-8 expression. The effects of TNF-α and GlyH-101 on IL-8 expression were additive and inhibited by AG1478. Inhibition of both CFTR and EGFR in HLMVECs significantly increased VEGF expression. The antioxidant N-acetyl cysteine significantly reduced ROS production and the increase in IL-8 and VEGF expression following CFTR inhibition.

**Conclusion:**

Functional endothelial CFTR limits oxidative stress and contributes to the normal anti-inflammatory state of HLMVECs. Therapeutic strategies to restore endothelial CFTR function in CF are warranted.

## Introduction

Cystic fibrosis (CF) lung disease is associated with neutrophilic airway inflammation, bronchiectasis, respiratory failure, and early mortality (Nichols and Chmiel, 2015). Defective or deficient expression of the cystic fibrosis transmembrane conductance regulator (CFTR) protein, an anion channel conducting mainly chloride and bicarbonate ions in airway epithelial cells (Saint-Criq and Gray, 2017), leads to dehydration of airway secretions with failure of mucociliary clearance and mucus plugging. In the early stages of CF lung disease this may lead to sterile inflammation (Zhou-Suckow et al., 2017), but inevitably the airways become chronically infected leading to further interleukin-8 (IL-8) driven neutrophilic inflammation (Nichols and Chmiel, 2015). In more severe disease, increased expression of vascular endothelial growth factor (VEGF) and associated peribronchial vascularity contributes to the immune and inflammatory responses in the CF airway, as well as the risk of hemoptysis (Martin et al., 2013).

A number of studies have indicated macro- and microvascular endothelial perturbation in people with CF (Romano et al., 2001; Solic et al., 2005; Poore et al., 2013; Rodriguez-Miguelez et al., 2016; Vizzardi et al., 2019) that may be associated with defective endothelial CFTR function (Noe et al., 2009; Brown et al., 2014; Peters et al., 2015; Totani et al., 2017). Increased circulating levels of von Willebrand factor (vWF) and tissue plasminogen activator (tPA), indicative of endothelial damage and altered hemostasis (Romano et al., 2001), and reduced flow-mediated dilation of the brachial artery, which was associated with more severe airway disease and symptomatic of reduced NO availability (Poore et al., 2013), were reported in CF. Hydrogen peroxide (H_2_O_2_)-induced oxidative stress and apoptosis required functional CFTR in human lung microvascular cells (HLMVEC), effects that were reversed by the CFTR inhibitor CFTRinh-172 (Noe et al., 2009). Reduced endothelial apoptosis in the vasculature of the CF lung has previously been reported which may be associated with reduced endothelial ceramide concentration (Noe et al., 2009). Endothelial CFTR plays an important role in maintaining hydration of the endothelial glycocalyx (Peters et al., 2015), the endothelial barrier function (Brown et al., 2014; Totani et al., 2017) and the availability of NO, while limiting IL-8 release (Totani et al., 2017).

Thus, together with reports of endothelial expression of CFTR in human umbilical vein endothelial cells (HUVEC) (Tousson et al., 1998; Totani et al., 2017), HLMVEC (Tousson et al., 1998), and human pulmonary artery endothelial cells (HPAEC) (Plebani et al., 2017; Totani et al., 2017), there is a growing body of evidence for expression and function of CFTR in the endothelium.

Previous studies have shown that functional CFTR at the epithelial cell surface is critical for limiting NF-κB mediated cell signaling leading to IL-8 expression both at baseline and following activation with pro-inflammatory cytokines IL-1 and TNF-α (Perez et al., 2007; Vij et al., 2009), for inhibiting epidermal growth factor receptor (EGFR)-mediated IL-8 (Kim et al., 2013) and VEGF synthesis (Martin et al., 2013) and for Nrf-2-mediated adaptive responses to oxidative stress (Chen et al., 2008).

The present study focuses on the pulmonary microvasculature and investigates the expression and function of CFTR as a regulator of oxidative stress, expression of the key transcriptional regulator of antioxidant defenses nuclear factor erythroid 2 [NF-E2]-related factor 2 (Nrf2), expression of pro-angiogenic VEGF and of the neutrophil chemoattractant IL-8, and reactive oxygen species (ROS)-mediated inflammatory cell signaling in HLMVECs grown on the basement membrane component, collagen IV.

The overall aim is to identify a role for dysfunctional endothelial CFTR in systemic inflammation and oxidative stress and, importantly, new therapeutic targets to reduce systemic inflammation and oxidative stress in people with CF.

## Materials and Methods

### Materials

All reagents were from Merck unless indicated otherwise.

#### Cell Culture

Human lung microvascular endothelial cells (HLMVECs) from non-smoking donors (Lonza Biologics) were maintained in complete EGM-2MV medium (EBM-2MV basal medium supplemented with 5% FBS, 0.04% hydrocortisone, 0.4% hFGF, 0.1% VEGF, 0.1% IGF-1, 0.1% ascorbic acid, 0.1% hEGF and 0.1% GA-100 (Lonza Biologics) and used at passage 5–8. The growth medium was changed 1 day after seeding and then every other day to 90% confluence. Cells were passaged using trypsin-EDTA to detach them, neutralized with warmed FBS and pelleted by centrifugation. The cell pellet was re-suspended in full growth medium (FGM) and seeded in multi-well plates (25 × 10^4^/6-well plate, 5 × 10^4^/24-well plate) coated with human collagen IV (0.1 mg/ml) and allowed to adhere overnight before treatment at the indicated times with the CFTR inhibitor GlyH-101 [5, 10, 20 μM (Friard et al., 2017)], dimethyl sulfoxide (DMSO, 0.1%) as vehicle control or TNF-α [10 ng/ml (Chen et al., 2008), Peprotech] for up to 24 h, which was established by cell viability testing in the presence of GlyH-101, as described below.

The specificity of GlyH-101 as a CFTR inhibitor was confirmed using inhibitors of other chloride ion channels. DCPIB (4-(2-butyl-6,7-dichlor-2-cyclopentyl-indan-1-on-5-yl) oxybutyric acid) (Tocris), a potent (IC_50_ 4.8 μM) and specific Volume-Regulated Anion Channel (VRAC) inhibitor was used at 20 μM (Friard et al., 2017), and Ani9 (2-(4-chloro-2-methylphenoxy)-N-[(2-methoxyphenyl)methylideneamino]-acetamide), a potent (IC_50_ 77 nM) and specific inhibitor of the calcium-activated chloride ion channel Transmembrane Member 16A (TMEM16A), also known as Anoctamin-1 (Ano-1), that lacks inhibitory activity against CFTR and VRAC (Seo et al., 2016), was used at 10 μM in HLMVEC cultures. HLMVECs were cultured in 5% CO_2_ at 37°C and used until passage 8.

In separate experiments, HLMVECs were treated with N-acetyl cysteine [NAC, 5 or 10 mM (Chen et al., 2008), as indicated] and the EGFR inhibitor AG-1478 [10 μM (Kim et al., 2013)] for 3 h, followed by incubation with GlyH-101 (20 μM) alone, and with TNF-α (10 ng/ml) for 16 and 24 h in FGM.

Human embryonic kidney cells (HEK-293) which do not express CFTR (Friard et al., 2017) and human bronchial epithelial cells (the human bronchial epithelial cell line 16HBE14o-) which do express CFTR (Cozens et al., 1994) were used as negative and positive controls, respectively. HEK-293 and 16HBE cells were cultured as described above in DMEM or MEM media, respectively, supplemented with 10% FBS, 1% penicillin-streptomycin, 1% L-glutamine. Cells were maintained until passage 30.

#### MTT Assay for Cell Viability

Human lung microvascular endothelial cells were seeded at a density of 1 × 10^4^ cells/well/100 μl of FGM in collagen-coated 96 well plates and left to adhere overnight at 37°C and 5% CO2. Next day the cells were treated with GlyH-101 and DMSO (0.1%) as vehicle control in FGM for 16, 24, and 48 h. In order to eliminate the effect of ascorbic acid in FGM on MTT color changes, the medium were replaced with 100 μl phenol red free medium (MEM) containing 0.3% of FBS per well. MTT 3-(4,5-dimethylthiazol-2-yl)-2,5-diphenyltetrazolium bromide (to a final concentration of 0.5 mg/ml) added to cells for 4 h at 37°C and 5% CO2 was reduced by cellular metabolic activity to insoluble formazan, which was solubilized with 100 μl 10% SDS in 0.01 M HCl overnight, and the absorbance read at 570 nm using a Spectramax i3x plate reader (Molecular Devices).

#### CFTR Activity Assay

Cystic fibrosis transmembrane conductance regulator activity was measured in HLMVECs, 16HBE and HEK-293 cells using the FLIPR Blue membrane potential assay (Molecular Devices), according to the manufacturer’s instructions, and as described by Maitra et al. (2013). Cells were seeded at 8 × 10^3^ cells per well and cultured overnight in collagen IV-coated optical-bottom black 96-well plates in 100 μl FGM. FLIPR Blue (100 μl) was directly added to the wells for 45 min at 37°C in FGM. Agonists, forskolin (100 μM)/IBMX (1000 μM), concentrations that significantly increase endothelial intracellular cAMP (Hopkins and Gorman, 1981; Sayner et al., 2004), were applied for 2 min and fluorescence measured with excitation 530 nm and emission 565 nm, using the Spectramax^®^ i3x fluorescent plate reader (Molecular Devices). GlyH-101 (20 μM) or DMSO (0.1%) control were applied 15 min before the agonists. Changes in membrane polarization are reported as changes in relative fluorescence units (ΔRFU). CFTR activity was detected as membrane depolarization at baseline, and in response to IBMX plus forskolin leading to increased uptake and fluorescence of intracellular FLIPR Blue, that could be inhibited by GlyH-101 (Maitra et al., 2013). Experimental conditions were tested in quadruplicate in each of three independent experiments.

#### Preparation of Cell Lysates for CFTR and Nrf2 Protein Assessment by Western Blot

Cells were lysed directly with 35 μl/well for 24-well (Nrf2) and 75 μl/well for 6-well (CFTR) plates of 1X sample buffer [7.5% (v/v) glycerol, 50 mM Tris-Base, 2% (w/v) sodium dodecyl sulfate (SDS), 100 mM DL-dithiothreitol (DTT), 0.01% (w/v) bromophenol blue, 2 mM MgCl*2*, 0.05% (v/v) benzonase nuclease, 2x protease inhibitor cocktail I], on ice for 10 min. Finally 1 μl of 0.25 M EDTA was added to each lysate and samples heated in a water bath at 50°C for 20 min.

#### Preparation of Cell Lysates for Phospho-c-Jun Protein Assessment by Western Blot

Human lung microvascular endothelial cells in 6-well plates were treated with DMSO (0.1%), GlyH-101 (20 μM) or TNF-α (10 ng/ml) for 30 min and the cells lysed in 100 μl/well lysis buffer [125 mM NaCl, 1 mM MgCl*2*, 20 mM Tris–HCl, 1% Triton X-100, Complete protease inhibitor cocktail (Roche) and phosphatase inhibitor (PhosSTOP, Roche)] at 4°C for 30 min. The cell lysates were centrifuged at 24000 RCF, at 4°C for 30 min and stored at −80°C. Cell lysates (30 μl) were mixed with 10 μl 4X sample loading buffer (30% v/v glycerol, 200 mM Tris–HCl pH 6.8, 8% w/v SDS, 400 mM DTT, 0.04% w/v bromophenol blue), and placed in a water bath (95°C) for 4 min.

#### Gel Electrophoresis and Western Blotting

Samples (35 μl) were loaded onto 7.5% (CFTR) or 10% (Nrf2, p-c-Jun) SDS-PAGE gels, with 5 μl of a molecular weight ladder with pre-stained proteins in the molecular size range 8–220 kDa (Sigma) or 10–250 kDa (Bio-Rad) loaded on each gel, and resolved proteins transferred using a *Trans-*Blot semi-dry transfer cell (Bio-Rad, United Kingdom) to 0.45 μm nitrocellulose membrane (Bio-Rad, United Kingdom). Membranes were blocked with 3% (w/v) skimmed milk powder in PBS (without Ca/Mg) with 0.1% Tween-20 (PBS-T), overnight at 4°C.

For CFTR, blots were incubated with 2 μg/ml “Mr. Pink” rabbit polyclonal antibody (kindly provided by Prof. Ineke Braakman, Utrecht University through the CFTR Folding Consortium, United States) diluted 1:500 in PBS-T. Blots were incubated with antibody for 1 h at room temperature (RT), washed and incubated with 0.05 μg/ml goat anti-rabbit-HRP (Dako) for 1 h at RT.

For Nrf-2, membranes were stained with 1 μg/ml rabbit polyclonal anti-Nrf2 (Santa Cruz) in blocking buffer for 1 h at RT, washed in PBS-T, and incubated with 0.125 μg/ml goat anti-rabbit-HRP (Dako) for 1 h at RT.

For p-c-Jun, membranes were stained with 0.2 μg/ml rabbit polyclonal anti-p-c-Jun [(Ser 63/73) Santa Cruz] overnight at 4°C in blocking buffer, and then 0.125 μg/ml goat anti-rabbit-HRP (DAKO) for 2 h at RT.

After washing in PBS, blots were incubated for 5 min in the dark with chemiluminescence substrate (Promega), followed by detection of the bands with ChemiDoc^TM^ MP System (Bio-Rad). The bands were normalized to β-actin in the samples, which was detected after stripping with Restore Western Blot Stripping Buffer (Thermo Fisher, United Kingdom) for 15 min and re-probing membranes with rabbit polyclonal anti-β-actin for 1 h at RT followed by 1 h incubation at RT with 0.05 μg/ml goat anti-rabbit HRP (DAKO). Bands were quantified using scanning densitometry and Quantiscan software.

#### Preparation of Supernatants and Cell Lysates for Quantification of IL-8, VEGF, SOD-2 and Catalase by ELISA

Supernatants from HLMVECs grown in 24-well plates were cleared by centrifugation (1500 RCF for 10 min at 4°C). Cells were lysed for 10 min on ice in hypotonic buffer (20 mM NaCl, 1% Triton X-100, and 20 mM Tris-base, pH 7.4), containing Complete protease inhibitors (Roche), and protease inhibitor cocktail I (Calbiochem). Lysates were centrifuged at 5000 RCF for 10 min at 4°C and samples stored at −80°C prior to analysis. IL-8 and VEGF were measured using Duo-Set ELISA kits (R&D Systems) according to the manufacturer’s instruction. Mitochondrial Mn-dependent superoxide dismutase (SOD-2) and catalase were quantified in neat cell lysates using ELISA kits (AbCam) according to manufacturer’s instructions, with standard curves prepared in the range 7.8–500 ng/ml.

#### Measurement of Intracellular ROS and H_2_O_2_

Human lung microvascular endothelial cells were seeded in 35 mm dishes for live cell imaging (25 × 10^4^ cells/dish), and HLMVECs and 16HBEs were seeded (8 × 10^3^/well/100 μl) for quantification of ROS in 96-well plates coated with collagen IV and incubated overnight. In some experiments, NAC (10 mM) was added for 3 h in FGM before adding the ROS detector, carboxy-H*2*DFFDA (5-(and-6)-carboxy-2′,7′-difluorodihydrofluorescein diacetate, (Invitrogen) in serum-free HBSS (+Ca/Mg) at 10 μM for 30 min. Cells were washed and incubated for 30 min in FGM, before being challenged with GlyH-101 and TNF-α for 5, 10, and 30 min in FGM. Changes in relative fluorescent units (ΔRFU) were compared with cells treated with 0.1% DMSO or media alone, as controls for GlyH-101 and TNF-α, respectively, and designated 100% at each time point. In some experiments, the mitochondrial targeting antioxidant MitoQ was added at 1 μM for 30 min in FGM prior to challenge of the cells with GlyH-101 for 5 min. Plates were read at 37°C in 5% CO*2*, Polar Star Optima plate reader, (BMG Labtech), with excitation at 485 nm and emission at 520 nm. For live cell imaging, dishes were examined on a LSM 710 confocal microscope (ZEISS) with immersion objectives and stage with temperature control, but without atmospheric control.

Hydrogen peroxide (H_2_O_2_) was measured in HLMVECs seeded at 5 × 10^4^ cells/well/300 μl FGM in wells of 24-well plates, incubated in the absence and presence of 10 μM diphenyleneiodonium chloride (DPI) in FGM for 1 h, before challenge with CFTR inhibitor GlyH-101 (20 μM) and control DMSO (0.1%) for 5, 10, 30 min. Cells were lysed in 125 μl of hypotonic lysis buffer [20 mM NaCl, 1% Triton X-100, 20 mM Tris-base, and pH to 7.4, with protease inhibitors (Calbiochem), and sodium azide (0.1% w/v), to inhibit catalase and peroxidase activity] and H_2_O_2_ quantified against an H_2_O_2_ standard in the range 3.125–200 μM using the Amplex Red assay (Chen et al., 2008).

#### Immunocytochemistry for CFTR, Nrf2, NF-κB and Phospho-c-Jun

HLMVECs, 16HBE or HEK293 cells in collagen-coated eight-well chamber slides were fixed in paraformaldehyde (4%) and permeabilized with Triton X-100 (0.1%). No primary antibody controls were included for each analysis.

For CFTR staining, non-specific antibody-binding sites were blocked with BSA (2%, for 16 h at 4°C) and HLMVECs, 16HBE or HEK293 cells incubated with rabbit anti-CFTR antibody “Mr. Pink” (1/500), followed by goat anti-rabbit antibody conjugated to Alexa Fluor-555 (2 μg/ml; Molecular Probes).

For NF-κB localization, non-specific antibody-binding sites were blocked with rabbit serum (5%, for 1 h) and HLMVECs incubated with mouse anti-p65 (4 μg/ml; Santa Cruz), for 16 h at 4°C, followed by rabbit anti-mouse antibody conjugated to Alexa Fluor-488 (2 μg/ml; Molecular Probes) for 1 h at RT.

For Nrf2 staining, HLMVECs were blocked with 5% goat serum overnight at 4°C, then rabbit polyclonal anti-Nrf2 (Santa Cruz) at 4 μg/ml was added for 1 h at 4°C, followed by Alexa Fluor-555 goat anti-rabbit IgG (2 μg/ml; Molecular Probes) for 1 h at RT.

For phospho-c-Jun staining HLMVECs were blocked with 5% goat serum overnight at 4°C, then rabbit anti-p-c-Jun [(Ser 63/73), Santa Cruz] at 0.8 μg/ml was added for 1 h at 4°C, followed by goat anti-rabbit Alexa Fluor-555 (Molecular Probes) at 2 μg/ml for 1 h at RT.

Nuclei were stained with 5 μg/ml Hoechst 33342 (Molecular Probes) for 10 min, and slides mounted in Fluor-Preserve Reagent for imaging with a LSM 710 confocal microscope (Zeiss).

#### RNA Extraction and Reverse Transcription

Total RNA was extracted using ReliaPrep RNA Cell Miniprep System (Promega) according to manufacturers’ instructions. The concentration and purity (absorbance ratios at 260/230 and 260/280 of ≥2.0) of RNA in samples was determined using a Nanodrop spectrophotometer. RNA was then reverse transcribed into cDNA using the first step of the GoTaq 2-Step RT-qPCR System (Promega) and either stored at −20°C or immediately used in quantitative PCR.

#### Quantitative Real-Time Polymerase Chain Reaction (qRT-PCR)

Cystic fibrosis transmembrane conductance regulator, IL-8 and β-actin transcripts were amplified from cDNA (1 μg), using the following primers: CFTR (105 bp) sense 5′- ATG CCC TTC GGC GAT GTT TT -3′and antisense 5′- TGA TTC TTC CCA GTA AGA GAG GC -3′;

IL-8 (194 bp) sense 5′- TTT TGC CAA GGA GTG CTA AAG A -3′ and antisense 5′- AAC CCT CTG CAC CCA GTT TTC -3′: and β-actin (93 bp) sense 5′- CGC GAG AAG ATG ACC CAG AT -3′ and antisense 5′- GCC AGA GGC GTA CAG GGA TA -3. The cycles for the qRT-PCR were: 2 min at 95°C, then 35 cycles for quantitation of IL-8 expression and 40 cycles for detection of CFTR mRNA of 10 s at 95°C, 20 s at 62°C, and 60 s at 72°C. Data were collected using a LightCycler 96 (Roche Diagnostics International Ltd.).

For nested or two-step, quantitative PCR, a 526 bp region of CFTR was first amplified using primers: sense 5′- ACA GCG CCT GGA ATT GTC AGA C -3′and antisense 5′- AGC GAT CCA CAC GAA ATG TGC C -3′, followed by a second round of amplification using the CFTR-specific primers and conditions used in the single-step quantitative PCR.

To exclude the possibility of amplification from contaminating genomic DNA during the qRT-PCR reaction, we utilized a DNAse step in the ReliaPrep protocol during mRNA extraction, and primers were designed to span at least one or more large (>20,000 bp) introns, precluding amplification from genomic DNA directly. Negative control reactions, not including template cDNA (no template control) or reverse transcriptase (“-RT,” or no amplification control), were also performed and accepted when the threshold value (Ct) was at least five cycles greater than the experimental amplifications. Measurements were performed in duplicate and accepted if the difference between the Ct values of the duplicates was less than 1. The generation of a single product of appropriate size was routinely checked by the presence of a single melt peak and by agarose gel electrophoresis. Data were analyzed using Roche LightCycler software. A relative expression method was implemented, normalizing the data by the internal control β-actin and expressing the final result relative to the control group.

#### Electroporation and CFTR Silencing

Electroporation were used to introduce small interfering RNA to target the CFTR gene in HLMVECs. HLMVECs (0.75 × 10*6*) were suspended in 100 μl of OptiMEM medium and transferred to a sterile cuvette for the NEPA21 electroporator (NepaGene) and 2 μl of siRNA for CFTR or validated non-targeting siRNA pool (50 μM) was added to a final concentration of 1 μM. For CFTR siRNA (ON-TARGET plus Human CFTR (1080) siRNA-SMART pool (Dharmacon) was used, which consists of a mixture of four siRNA with target sequence as the following:

1-GAACACAUACCUUCGAUAU
2-GUACAAACAUGGUAUGACU
3-GUGAAAGACUUGUGAUUAC
4-GCAGGUGGGAUUCUUAAUA

The cells were electroporated according to the pulse settings in Table 1.

**TABLE 1:** Electroporation pulse settings.

Poring pulse						Transfer pulse					
V	Length ms	Interval ms	No.	D. Rate (%)	Polarity	V	Length ms	Interval ms	No.	D. Rate (%)	Polarity
170	2.5	50	2	10	+	20	50	50	5	40	±

After electroporation cells were transferred to the wells of a collagen-coated 6 well plate at a final concentration of 1.5 × 10*6* cell/well/2 ml of FGM. The medium then was changed every 24 h, for a total of 72 h, then the supernatants for the last 24 h were harvested and processed and used to analyze IL-8 concentrations by ELISA.

#### Statistical Analysis

**FIGURE 1:**
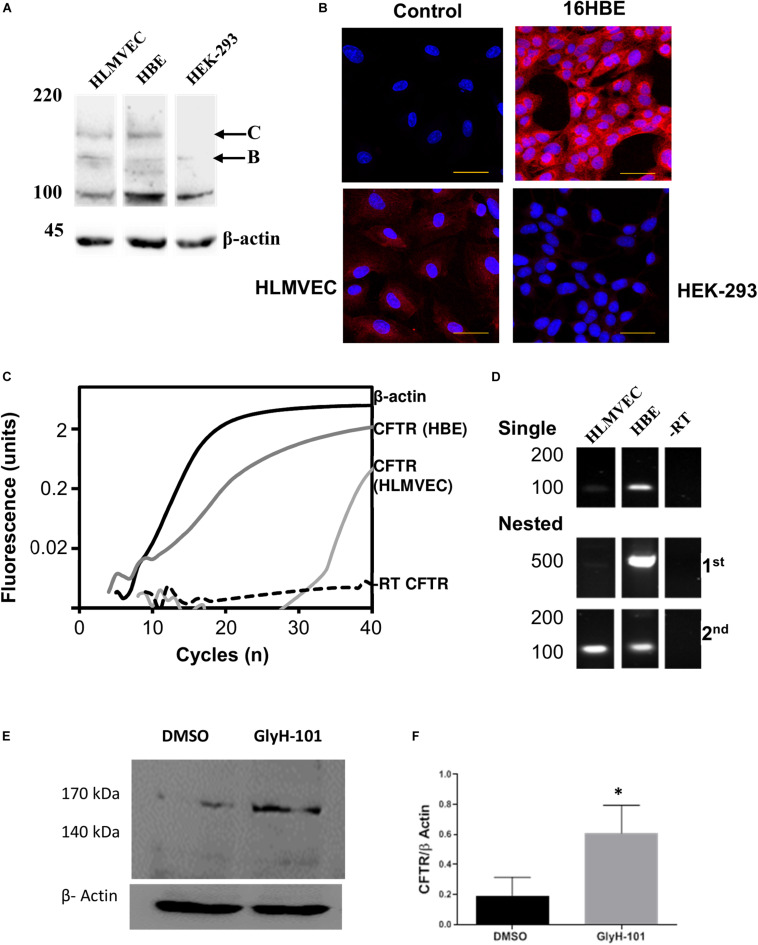
CFTR expression in HLMVECs. **(A)** Immunoblot of CFTR in whole cell extracts of HLMVEC, 16HBE and HEK-293 cells. All data are representative of that obtained in at least three independent experiments. **(B)** Immunolocalization of CFTR in HLMVEC, 16HBE and HEK-293 cells (the control refers to the no-primary antibody negative control). All images were acquired and displayed under identical conditions. **(C)** RT-PCR amplification of CFTR (light gray), β-actin (solid) and reverse-transcription negative control (-RT CFTR, dotted line) in HLMVECs, and CFTR in 16HBE cells (positive control, dark gray). **(D)** Gel analysis of CFTR cDNA amplified from HLMVEC, 16HBE mRNA and -RT control following single and nested RT-PCR. **(E,F)** CFTR expression in HLMVECs cell lysate by western blot after 16 h incubation with GlyH-101 (20 μM) and DMSO (0.1%) vehicle control. Data were normalized to β-actin, numbers expressed as average of three independent experiments. Each experiment was conducted on HLMVECs obtained from three different donors (^∗^*p* < 0.05 for the difference between GlyH-101 and control). The scale bar represents 50 μm.

Statistical analysis was carried out using GraphPad Prism version 8. Data is presented graphically as mean ± SEM, and analyzed by one-way or two-way ANOVA with two-tailed *post hoc* tests for multiple comparisons as appropriate and as indicated in the Figure legends. A directional one-tailed *t*-test was applied in the case of the hypothesized increase in CFTR expression following inhibition of CFTR activity (Figure 1F). Multiplicity adjusted *p* values are given in the text to four decimal places.

The relative effect size, Cohen’s d, was determined by calculating the mean difference between two groups and dividing the result by the pooled standard deviation. Cohen’s *d* = (*M*_2_−*M*_1_)/*SD*_pooled_, where:

*SD*_pooled_ = √((*SD*_1_^2^ + *SD*_2_^2^)/2), and Cohen’s *d* > 0.8 is considered a large effect size.

## Results

### CFTR Expression

Western blotting and RT-PCR were used to confirm CFTR expression in HLMVEC under the cell culture conditions used in these experiments, including growth on collagen IV coated cultureware. CFTR could be detected on western blot as two high molecular weight bands in HLMVEC lysates, the partially glycosylated band B (140 kDa) and fully mature band C (170 kDa) (Figure 1A). However, the level of expression was highly variable between preparations. In separate experiments, the same CFTR protein bands were detected in 16HBE, but not in HEK293 cells. Levels of CFTR detected by IHC appeared to be lower in HLMVECs than in the 16HBE bronchial epithelial cell line and, in addition to the plasma membrane, CFTR was detected in association with intracellular organelles possibly the endoplasmic reticulum around the nucleus (Figure 1B). Additionally, expression of CFTR mRNA was detected in HLMVECs (ΔCT = 25.35 ± 0.55, *n* = 3), although at much lower levels that in 16HBEs (ΔCT = 3.78 ± 0.53, *n* = 3; *p* < 0.0001), when normalized to housekeeping β-actin expression at threshold of 0.02 ΔRFU (Figure 1C). The expression of CFTR mRNA In HLMVECs was confirmed by nested PCR (Figure 1D) which increased the intensity of the expected 100 bp CFTR product observed in single round PCR while the expected 500 bp product was faintly detected.

In view of previous reports of the negative regulation of CFTR expression by CFTR activity (Wang et al., 2016), we tested the hypothesis that CFTR inhibition would increase CFTR expression. Indeed, the abundance of CFTR in cells treated for 16 h with GlyH-101 (20 μM) was significantly higher (*p* = 0.035, one-tailed *t*-test) with a large effect size (Cohen’s *d* = 1.55) compared to control cells treated with DMSO (0.1%) (Figures 1E,F).

### GlyH-101 and HLMVEC Viability

**FIGURE 2:**
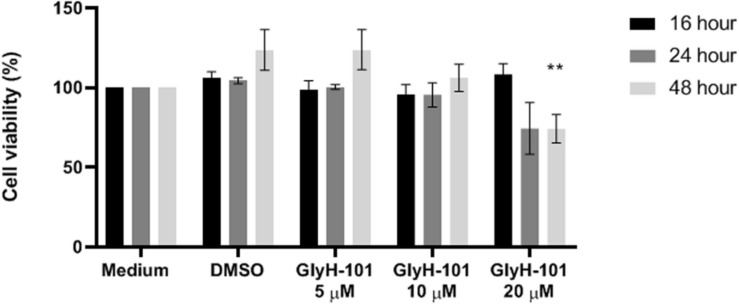
Effect of GlyH-101 on HLMVEC viability. HLMVECs were grown in FGM medium alone (medium), or treated for 16, 24, and 48 h with CFTR inhibitor GlyH-101 at 5, 10, and 20 μM or 1% DMSO as vehicle control. Results were normalized for the cells grown in medium alone, and data for the effect of GlyH-101 compared with DMSO (0.1%) vehicle control data. Data were expressed as mean ± SEM (**p value < 0.01 comparing GlyH-101 (20 μM) to DMSO (0.1%) at 48 h, *n* = 3 independent experiments). Data were analyzed with two-way ANOVA and Tukey’s multiple comparison test.

In order to investigate the effect of the CFTR inhibitor and the vehicle control on metabolic activity as a measure of cell cytotoxicity, cells were grown in medium (FGM) alone, or incubated with GlyH-101 (5, 10, 20 μM) or DMSO (0.1%), as the vehicle control, for the times indicated (Figure 2) and metabolic activity measured using the MTT assay. Data was normalized to the metabolic activity of cells grown in medium alone. Incubation of cells with GlyH-101 at concentrations of 5–20 μM for up to 24 h had no significant effect on HLMVEC metabolic activity (MTT reduction) compared to the vehicle control (Figure 2). After 48 h incubation with the highest (20 μM) GlyH-101 concentration, the significant (*p* = 0.002) decrease in MTT reduction (74.2 ± 15.4%) compared with the DMSO control (123.6 ± 22.0%) was associated with a reduction in the number of cells adherent to the plate. Experiments using GlyH-101 to inhibit CFTR were therefore conducted over periods of up to 24 h.

### CFTR Activity

**FIGURE 3:**
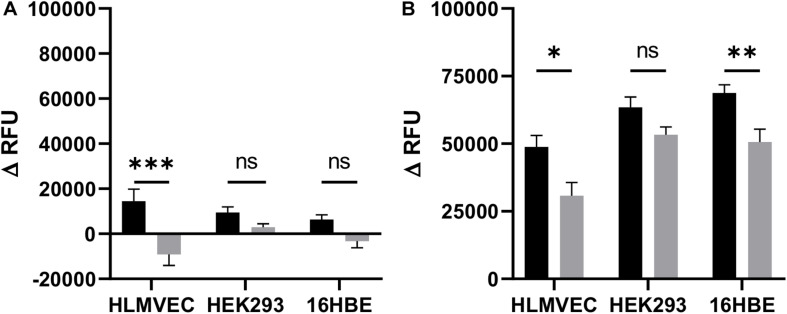
CFTR activity in HLMVECs at baseline and following cAMP-dependent activation. CFTR activity was measured at baseline **(A)** and following cAMP-dependent activation **(B)** of HLMVECs, non-CFTR expressing HEK-293 cells and CFTR expressing 16HBE cells for comparison. CFTR activity at baseline was measured as a decrease in relative fluorescence units (ΔRFU) following treatment of cells with the CFTR inhibitor GlyH-101 (20 μM, gray bars), reflecting membrane hyperpolarization and decreased uptake of FLIPR compared to cells treated with 0.1% DMSO vehicle control (black bars). Activation of CFTR **(B)** was measured as an increase in fluorescence (ΔRFU) following membrane depolarization of HLMVECs, HEK-293, and 16HBE cells activated with forskolin (100 μM) plus IBMX (1000 μM) (F/I) for 2 min in the absence (black bars) and presence (gray bars) of GlyH-101 (*n* = 3 independent experiments, each carried out in quadruplicate). Data were analyzed by two-way ANOVA and Holm-Sidak multiple comparisons test (^∗^*p* < 0.05, ^∗∗^*p* < 0.01, ^∗∗∗^*p* < 0.001 for the comparisons indicated).

Cystic fibrosis transmembrane conductance regulator activity was detected as membrane depolarization at baseline, and in response to IBMX plus forskolin leading to increased uptake and fluorescence of intracellular FLIPR Blue, that could be inhibited by GlyH-101 (Maitra et al., 2013). The significant (*p* = 0.0002) inhibitory effect (Cohen’s *d* = 1.32) of GlyH-101 on control cells indicates constitutive activity of CFTR in HLMVECs (Figure 3A). This was not seen in HEK293 cells used as a negative control or 16HBE cells used as a positive control. The cAMP-elevating agent forskolin (an adenylate cyclase activator) together with IBMX (a cAMP-phosphodiesterase inhibitor) strongly (*p* < 0.0001) stimulated HLMVEC membrane depolarization in all cells (Figure 3B, black bars) compared to baseline (Figure 3A, black bars). CFTR activity was measured as the increase in membrane depolarization (increase in Δ RFU) that was significantly inhibited by GlyH-101 (20 μM) (Figure 3B, gray bars). In HLMVEC, GlyH-101 significantly (*p* = 0.0495) inhibited membrane depolarization by 34% (Cohen’s *d* = 1.17). The depolarization response of 16HBE cells was significantly (*p* = 0.0095) greater than that of HLMVECs and was significantly (*p* = 0.0068) inhibited 26.4% (Cohen’s *d* = 1.08) by GlyH-101. This decrease in fluorescence was attributed to inhibition of CFTR activity. HEK293 cells that do not express CFTR (Friard et al., 2017) showed a greater depolarization in response to forskolin/IBMX than HLMVECs, but there was no significant inhibitory effect of GlyH-101 (Figure 3B) and no effect on unstimulated control cells (Figure 3A).

### CFTR and Oxidative Stress

**FIGURE 4:**
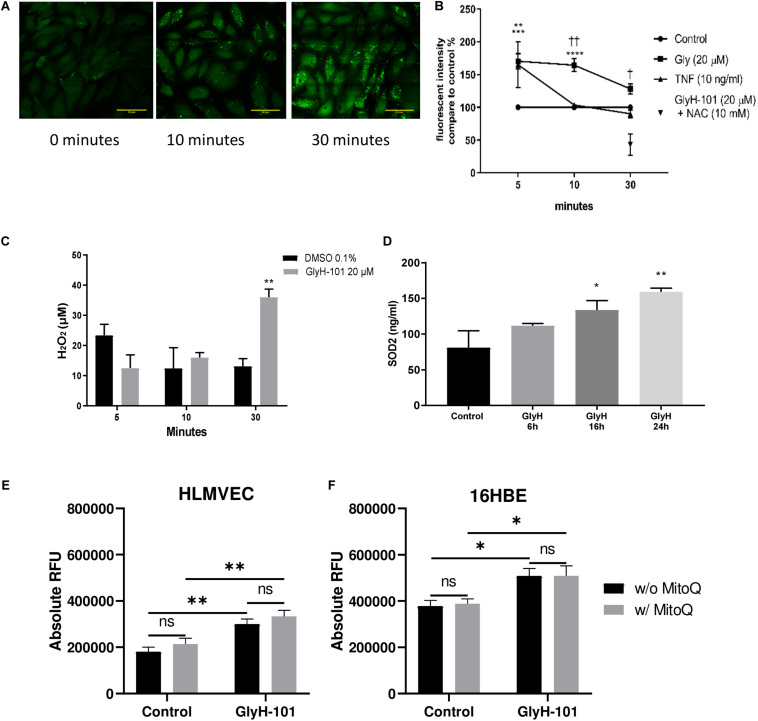
Oxidative stress in HLMVECs following CFTR inhibition with GlyH-101. **(A)** Intracellular ROS in HLMVECs using live cell imaging. Intracellular ROS in HLMVECs detected using carboxy H2-DFFDA. GlyH-101 (20 μM) was added and the fluorescence detected at 0, 10, and 30 min. Scale bar 50 μm. Images representative of duplicate wells in a single experiment. **(B)** Quantification of intracellular ROS in HLMVECs using carboxy-H2DFFDA. HLMVECs were incubated for 5–30 min in the absence and presence of GlyH-101 (20 μM) and TNF-α (10 ng/ml) and ROS detected by intracellular DFFDA. The experiments were conducted in triplicate, *n* = 6 independent experiments for GlyH-101 in the absence and presence of 10 mM NAC, and *n* = 3 for TNF-α. Control values at each time point were designated 100% (filled circles). Data points represent the mean ± SEM. Results were analyzed with two-way ANOVA and Tukey’s multiple comparisons test. (**** *p* value < 0.0001, *** *p* value < 0.001) comparing GlyH-101 to control and (** *p* value < 0.01) comparing TNF-α to control, (††, *p* value < 0.01, † *p* value < 0.05 comparing GlyH-101 and TNF-α). **(C)** Quantification of intracellular H_2_O_2_ using Amplex Red. HLMVECs were treated with GlyH-101 for 5–30 min, and H_2_O_2_ measured in cell lysates using Amplex Red reagent. ** *p* value < 0.01 in comparison to DMSO after 30 min, (*n* = 4). Data were expressed as average ± SEM and analyzed with two way ANOVA and Tukey’s *post hoc* test. **(D)** Intracellular SOD2. SOD2 was measured by ELISA in HLMVECs cell lysates following CFTR inhibition with GlyH-101 (20 μM). Data were analyzed with one way ANOVA and Dunnett’s test, *n* = 3 (* *p* < 0.05, ** *p* value < 0.01 compared to DMSO control at 24 h). **(E)** Quantification of intracellular ROS in HLMVECs using carboxy-H2DFFDA. HLMVECs were incubated for 30 min with/without MitoQ (1 μM) followed by 5 min incubation with GlyH-101 (20 μM) and ROS detected by intracellular DFFDA. The experiments were conducted in quadruplicate, *n* = 3 independent experiments. (** *p* value < 0.01 for the comparisons indicated). Data were analyzed by two-way ANOVA with Tukey’s multiple comparison *post hoc* test. RFU, relative fluorescence units. **(F)** Quantification of intracellular ROS in 16HBEs using carboxy-H2DFFDA. 16HBEs were incubated for 30 min with/without MitoQ (1 μM) followed by 5 min incubation with GlyH-101 (20 μM) and ROS detected by intracellular DFFDA. The experiments were conducted in quadruplicate, *n* = 3 independent experiments. (* *p* < 0.05 for the comparisons indicated). Data were analyzed by two-way ANOVA with Tukey’s multiple comparison *post hoc* test. RFU, relative fluorescence units.

In view of previous reports of the effect of dysfunctional epithelial CFTR on increased intracellular H_2_O_2_ (Chen et al., 2008), we investigated the effect of CFTR inhibition on the generation of intracellular ROS and, specifically, H_2_O_2_ levels. Using live-cell imaging, low levels of intracellular ROS were observed at time zero, which increased over 30 min after treating the cells with GlyH-101 (Figure 4A). However, using a quantitative approach to measuring ROS generation, a faster response was detected in stimulated cells compared to unstimulated control cells (Figure 4B). The slower response during live cell imaging may reflect the absence of atmospheric control during the experiment.

Both TNF-α, included as a positive control, and GlyH-101 induced a significant (*p* = 0.0012 and *p* = 0.0004, respectively) increase in ROS of 165.2 ± 35.1% and 170 ± 11.5% compared to unstimulated control cells at 5 min. The response to GlyH-101 was sustained over 30 min and was significantly higher than the response to TNF-α at 10 and 30 min (*p* = 0.003 and *p* = 0.0173, respectively), while the response to TNF-α was back to control levels at 10 min (Figure 4B).

N-acetyl cysteine (10 mM), added 3 h before GlyH-101, had no effect on ROS production at 5 and 10 min following stimulation with GlyH-101. After 10 min, ROS production in response to GlyH-101 was 164.5 ± 9.9% of the control value, and in the presence of NAC was still 156.9 ± 50% of the control value (*n* = 6). However, NAC (10 mM) not only reversed the effect of GlyH-101 on ROS levels at 30 min, but also significantly (*p* = 0.005) reduced the level of ROS to 42.8 ± 28.1% fluorescence intensity, below that observed in control cells (Figure 4B).

H2DFFDA detects intracellular oxidative stress rather than a specific reactive species (Eruslanov and Kusmartsev, 2010). We therefore measured H_2_O_2_, one of the major ROS in cells. H_2_O_2_ was not detected in supernatants, but intracellular H_2_O_2_ was significantly (*p* = 0.0022) increased (Cohen’s *d* = 4.39) following 30 min treatment with GlyH-101 (36.1 ± 2.7 μM) compared to control (13.2 ± 2.5 μM), with no significant changes at earlier time points (Figure 4C). Incubation of cells with 10 μM DPI for 1 h prior to and during GlyH-101 challenge had no effect on H_2_O_2_ concentrations.

Intracellular SOD-2 protein levels were significantly increased at 16 h (*p* = 0.05, Cohen’s *d* = 1.58) and at 24 h (*p* = 0.0054, Cohen’s *d* = 2.63) following CFTR inhibition (Figure 4D). No significant changes in catalase protein levels were observed over 24 h (data not shown).

The staining pattern observed during live cell imaging of ROS was punctuate (Figure 4A), which suggested a mitochondrial source of ROS. However, the mitochondrial-targeting antioxidant MitoQ had no effect in HLMVEC or 16HBE cells on ROS production, measured as a change in ΔRFU of carboxy-H2DFFDA over 5 min following challenge with GlyH-101 (Figures 4E,F). GlyH-101 induced a significant increase in ROS in HLMVECs in the absence (*p* = 0.0043, Cohen’s *d* = 1.65) and presence (*p* = 0.0042) of MitoQ, and in the 16HBE bronchial epithelial cell line in the absence (*p* = 0.0275, Cohen’s *d* = 1.3) and presence (*p* = 0.0474) of MitoQ, which had no significant effect.

### CFTR and Nrf-2 Expression

**FIGURE 5:**
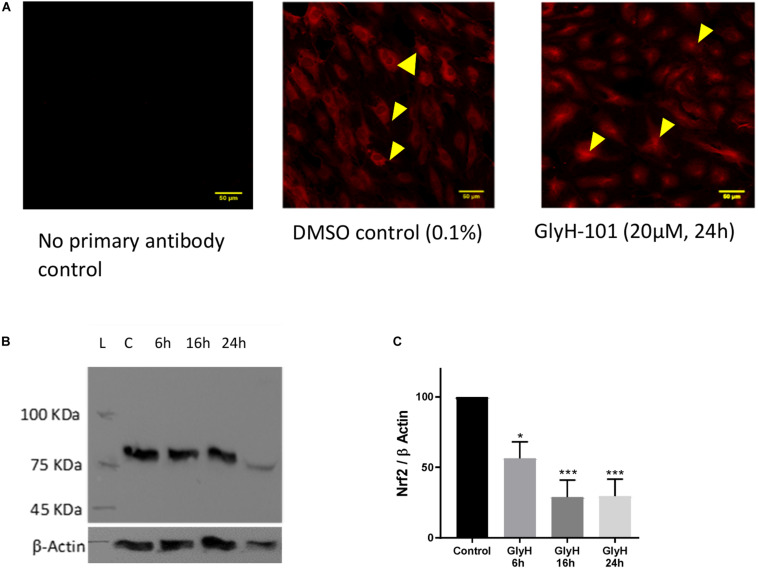
The effect of CFTR inhibition on Nrf-2 expression. The effect of CFTR inhibition on Nrf2 expression in HLMVECs over 24 h was investigated by immunocytochemistry **(A)** and by western blot **(B,C)**. **(A)** CFTR inhibition by GlyH-101 (20 μM) results in loss of cytosolic Nrf2 (arrows in control) with residual Nrf2 in nuclei. Scale bar 50 μm. Images are representative of two independent experiments. **(B,C)** Nrf2 was detected on western blot as an 85 kDa band in control cells (lane C) at 24 h **(B)**, this value was significantly reduced by 70% with GlyH-101 over 24 h **(C)**. Bars indicate mean ± SEM (* *p* < 0.05, *** *p* value < 0.001) for comparison with 24 h control, *n* = 3 independent experiments. Data were analyzed by one-way ANOVA with Dunnett’s *post hoc* test.

Chen et al. (2008) reported significantly reduced Nrf2 expression in CF epithelia and in normal epithelial cells treated with the CFTR inhibitor CFTR_inh_-172 (Chen et al., 2008). Therefore, Nrf2 expression in HLMVECs was investigated following CFTR inhibition with GlyH-101 (20 μM) for up to 24 h. IHC demonstrated that Nrf2 was mostly absent from the cytoplasm after 24 h incubation with GlyH-101, compared to control cells (Figure 5A). The overall abundance of Nrf2 was significantly (*p* = 0.0425) decreased at 6 h and had decreased further still at 16 h (*p* = 0.0032) and by 70.8 ± 11.6% at 24 h, (*p* = 0.0034, Cohen’s *d* = 4.82), compared to control cells (Figures 5B,C).

### CFTR and VEGF Expression

**FIGURE 6:**
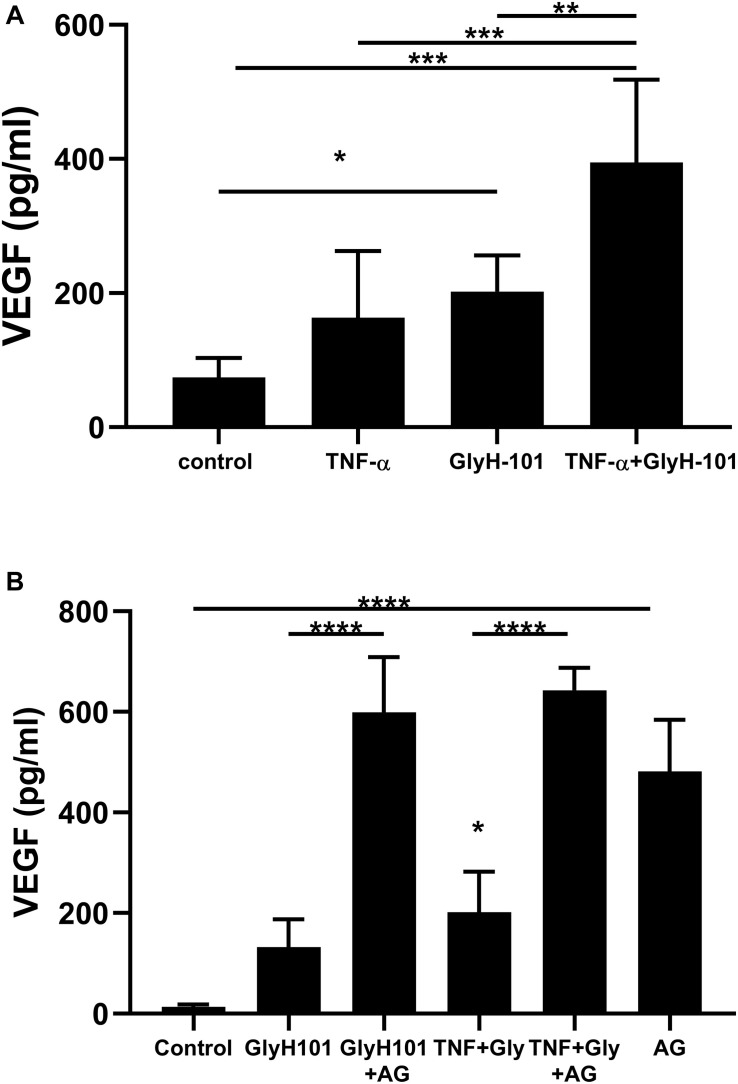
The effect of CFTR inhibition on VEGF concentrations in HLMVEC culture supernatants. **(A)** VEGF concentration after 16 h CFTR inhibition with GlyH-101 (20 μM), in the absence and presence of TNF-α (10 ng/ml). VEGF was measured by ELISA, and concentrations expressed as average ± SEM (* *p* value < 0.05, ** *p* value < 0.01, *** *p* value < 0.001 for the comparisons indicated). Data were analyzed with one-way ANOVA test and Tukey’s multiple comparison test, *n* = 6 independent experiments, each carried out in triplicate. **(B)** The effect of AG1478 on VEGF concentration alone, and after 24 h CFTR inhibition with GlyH-101 (20 μM) in the absence and presence of TNF-α. VEGF was measured by ELISA, and concentrations expressed as average ± SEM (**** *p* value < 0.0001 for the comparisons indicated, * *p* value < 0.05 for TNF + Gly compared to the control value). Data were analyzed with one-way ANOVA test and Tukey’s multiple comparison test, *n* = 3 independent experiments, each carried out in triplicate.

Since ROS induce the expression of endothelial VEGF (Kim and Byzova, 2014), we investigated the effect of GlyH-101 on VEGF levels in HLMVEC culture supernatants. Initial experiments established that GlyH-101 (5, 10, 20 μM) increased endothelial VEGF expression over 16 h, with a significant effect at 20 μM GlyH-101 (Figure 6), but not at lower concentrations (data not shown). GlyH-101 (20 μM) significantly (*p* = 0.0375, Cohen’s *d* = 2.95) increased VEGF concentration in the supernatants (202.1 ± 22.1 pg/ml) compared to control (74.24 ± 11.8 pg/ml) over 16 h (Figure 6A). TNF-α, alone, did not have a significant effect on VEGF levels, but further significantly (*p* = 0.0019, Cohen’s *d* = 2.01) increased the VEGF response to GlyH-101 to 394.5 ± 50.4 pg/ml. Treatment of cells with NAC (5 mM) significantly (*p* = 0.0056) reduced VEGF levels in the presence of GlyH-101 plus TNF-α to 105.6 ± 21.8 pg/ml, which was thus found not significantly higher than control levels (data not presented graphically).

In cultured airway epithelial cells, treatment with CFTR inhibitors triggered EGFR phosphorylation and activation that was required for VEGF synthesis (Martin et al., 2013). We therefore investigated the effect of EGFR inhibition on VEGF synthesis in HLMVECs. Pre-treatment with the EGFR tyrosine kinase inhibitor AG1478 (10 μM) caused a non-significant increase in VEGF levels at 16 h (results not shown). However, a significant (*p* < 0.0001) increase in VEGF (481.6 ± 59.2 pg/ml) was seen following EGFR inhibition with AG1478, alone, compared to control (13.48 ± 2.9 pg/ml) over 24 h (Figure 6B). Although GlyH-101 alone had no significant effect on VEGF synthesis over 24 h, AG1478 increased VEGF synthesis in the presence of GlyH-101 (*p* < 0.0001), and in the presence of both the CFTR inhibitor and EGFR inhibitor VEGF levels were 598.8 ± 63.7 pg/ml. Addition of TNF-α to GlyH-101 at this time point induced a small significant (*p* = 0.046) increase in VEGF levels, and addition of AG1478 further significantly (*p* < 0.0001) increased VEGF levels in the presence of GlyH-101 plus TNF-α.

### CFTR and IL-8 Expression

**FIGURE 7:**
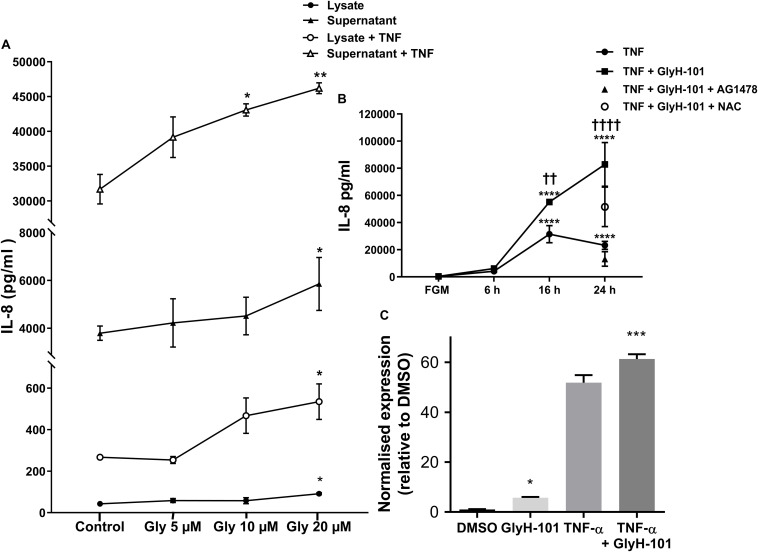
IL-8 in HLMVEC cells and supernatants. **(A)** Concentration response curve for CFTR inhibitor GlyH-101 in the absence and presence of TNF-α (10 ng/ml) for 16 h. GlyH-101 at 20 μM significantly increased IL-8 in supernatant, cell lysate, with and without TNF-α (* *p* < 0.05, ** *p* < 0.01) comparing GlyH-101 with control for each condition using one-way ANOVA (*n* = 3). **(B)** Time course for the effect of TNF-α (10 ng/ml) alone and in the presence of GlyH-101 (20 μM) on IL-8 levels in HLMVEC supernatants. (**** *p* < 0.0001 compared to 24 h control, FGM), (†† value < 0.01, † † †† value < 0.0001 compared to TNF-α alone), (*n* = 3). AG1478 (10 μM, filled triangle) and NAC (5 mM, open circle) significantly (*p* < 0.01 and *p* < 0.05, respectively) inhibited the response to the combination of TNF-α plus GlyH-101 at 24 h. Numbers were expressed as mean ± SEM. The data were analyzed with two-way ANOVA and Tukey’s multiple comparison test, or one-way ANOVA for the effects of AG1478 and NAC on the response to the combination of TNF-α plus GlyH-101. **(C)** Relative changes in IL-8 mRNA levels normalized to β-actin (2-delta delta Ct values), following 16 h incubation with GlyH-101 (20 μM) and TNF-α (10 ng/ml), alone and together, compared to DMSO (0.1%) control, *n* = 3. (* *p* < 0.05 comparing GlyH-101 with DMSO control, and *** *p* < 0.001 comparing TNF-α + GlyH-101 with TNF-α alone).

Functional CFTR was reported to limit bronchial epithelial expression of IL-8 (Perez et al., 2007). We therefore investigated the effect of CFTR inhibition on IL-8 expression by HLMVECs. Control, unstimulated, HLMVECs expressed IL-8 that was predominantly in soluble form and released into supernatants (Figure 7A). The CFTR inhibitor GlyH-101 (20 μM) significantly increased IL-8 protein levels in both supernatants (*p* = 0.0359) and HLMVEC cell lysates (*p* = 0.0234) (Figure 7A). This effect was not seen with GlyH-101 added at 5 μM and 10 μM. IL-8 concentrations were significantly (*p* < 0.0001) higher in supernatants than lysates under all conditions. TNF-α (10 ng/ml) further significantly (*p* < 0.0001) increased IL-8 levels in the presence of GlyH-101 (20 μM) in both fractions over 16 h. In the presence of TNF-α, GlyH-101 (20 μM) significantly increased IL-8 levels in cell lysates (*p* = 0.0193) and supernatants (*p* = 0.0047) compared to TNF-α alone.

Over a 24-h time course, TNF-α (10 ng/ml) and the combination of TNF-α (10 ng/ml) plus GlyH-101 (20 μM) significantly (*p* < 0.0001) increased supernatant IL-8 levels at both 16 h and 24 h (Figure 7B). The response to TNF-α alone plateaued at 16 h (Figure 7B), but the combination of GlyH-101 plus TNF-α significantly increased IL-8 levels at 16 h (*p* = 0.0043) and at 24 h (*p* < 0.0001) compared to the effect of TNF-α alone.

AG1478 (10 μM) and NAC (5 mM) significantly (*p* = 0.0028 and *p* = 0.0444, respectively) inhibited the response to the combination of TNF-α plus GlyH-101 at 24 h.

The increase in protein concentration mirrored the increase in IL-8 mRNA expression following 16 h incubation with GlyH-101 (20 μM) and TNF-α (10 ng/ml), alone and in combination (Figure 7C). The normalized relative expression of IL-8 mRNA was significantly (*p* = 0.0258, Cohen’s *d* = 17.82) increased in the presence of GlyH-101 alone. GlyH-101 also further significantly (*p* = 0.0004, Cohen’s *d* = 3.72) enhanced the response to TNF-α.

### Specificity of GlyH-101

Melis et al. (2014) reported non-specific inhibitory effects of GlyH-101 on volume-sensitive outwardly rectifying chloride conductance (VSORC), also termed volume-regulated anion channel current (VRAC), and calcium-activated chloride conductance (CaCC) in murine cell lines expressing CFTR (Melis et al., 2014). More recently, Friard et al. (2017) also reported inhibitory effects of GlyH-101 on VRAC conductance in the immortalized human embryonic kidney cell line, HEK-293 cells, with an IC_50_ of 9.5 μM and 80% inhibition at 20 μM (Friard et al., 2017).

**FIGURE 8:**
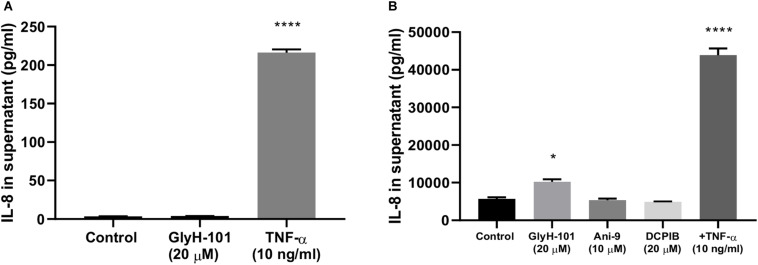
CFTR-specific inhibition mediates pro-inflammatory effects in HLMVECs. **(A)** non-CFTR expressing HEK-293 cells were incubated with GlyH-101 (20 μM) and TNF-α (10 ng/ml) as a positive control, and IL-8 was measured in culture supernatants by ELISA (*n* = 3) (**** *p* < 0.0001 compared to control). **(B)** HLMVECs were cultured for 24 h in the absence and presence of the CFTR inhibitor GlyH-101 (20 μM), the calcium-activated chloride ion channel Transmembrane Member 16A (TMEM16A)-specific inhibitor Ani-9 (10 μM), the Volume-Regulated Anion Channel (VRAC)-specific inhibitor DCPIB (20 μM) and TNF-α (10 ng/ml) as a positive control, and IL-8 was measured in culture supernatants by ELISA (*n* = 3). Data were analyzed by one-way ANOVA with Dunnett’s multiple comparisons *post hoc* test. (* *p* < 0.05 comparing GlyH-101 with control, and **** *p* < 0.0001 comparing TNF-α with control).

HEK-293 cells, unlike endothelial cells, do not express CFTR or the calcium-activated chloride ion channels anoctamin-1 and anoctamin-2 (ANO1/2, also termed TMEM16A and TMEM16B, respectively) (Friard et al., 2017). Therefore to investigate whether the pro-inflammatory effects of GlyH-101 effects on HLMVEC were due to inhibitory effects on VRAC activity, HEK-293 cells were incubated with 20 μM GlyH-101 for 24 h and IL-8 levels measured in cell culture supernatants. Because HEK-293 cells express moderate levels of endogenous TNFα receptors (Greco et al., 2015), TNFα (10 ng/ml) was used as a positive control. TNFα (10 ng/ml) significantly (*p* < 0.0001, Cohen’s *d* = 42.1) increased IL-8 concentrations in HEK-293 cell culture supernatant compared to control levels (Figure 8A), whereas GlyH-101 had no effect, indicating that effects of GlyH-101 on IL-8 expression are not mediated by VRAC.

This was confirmed using the potent (IC_50_ 4.8 μM) and specific VRAC inhibitor DCPIB (4-(2-butyl-6,7-dichlor-2-cyclopentyl-indan-1-on-5-yl) oxybutyric acid (Friard et al., 2017), in cultures of HLMVECs with GlyH-101 (20 μM) and TNFα (10 ng/ml) as positive controls. Both TNFα (*p* < 0.0001, Cohen’s *d* = 16.73) and GlyH-101 (*p* = 0.017, Cohen’s *d* = 4.7) significantly increased IL-8 in culture supernatants, but DCPIB at 20 μM had no effect (Figure 8B).

Additionally, the potent (IC_50_ 77 nM) TMEM16A-specific inhibitor Ani9 (2-(4-chloro-2-methylphenoxy)-N-[(2-methoxyphenyl)methylideneamino]-acetamide), that lacks inhibitory activity against CFTR and VRAC (Seo et al., 2016) did not induce significant IL-8 expression by HLMVECs at the relatively high concentration of 10 μM (Figure 8B).

### Effect of CFTR Gene Silencing

**FIGURE 9:**
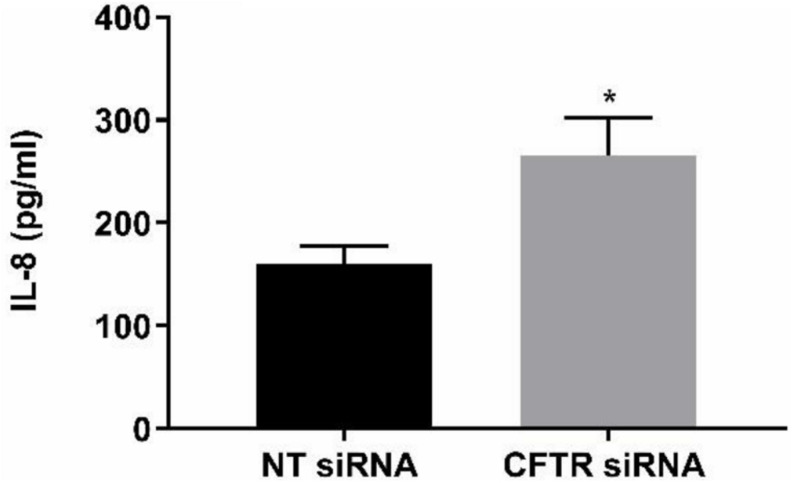
CFTR gene silencing. CFTR gene silencing using siRNA in HLMVECs induced a significant increase in IL-8 in supernatants. After targeting CFTR mRNA for 72 h, the results were compared with non-targeting siRNA as negative control. (*p < 0.05, *n* = 4 independent experiments, with each experiment carried out in duplicate). Data were expressed as mean ± SEM and analyzed by *t*-test.

The anti-inflammatory function of endothelial CFTR was confirmed using a siRNA approach to CFTR gene silencing. A significantly (*p* = 0.041, Cohen’s *d* = 1.84) higher concentration of IL-8 was measured in the supernatants of HLMVECs transfected with CFTR siRNA compared with cells transfected with non-targeting siRNA. The non-targeted cells yielded 160.01 ± 17.2 pg/ml IL-8 in HLMVEC supernatants, while silencing of CFTR (siCFTR) in HLMVECs gave 265.4 ± 36.7 pg/ml IL-8 in the supernatant (Figure 9) confirming constitutive CFTR activity in HLMVECs.

### CFTR and Inflammatory Cell Signaling

**FIGURE 10:**
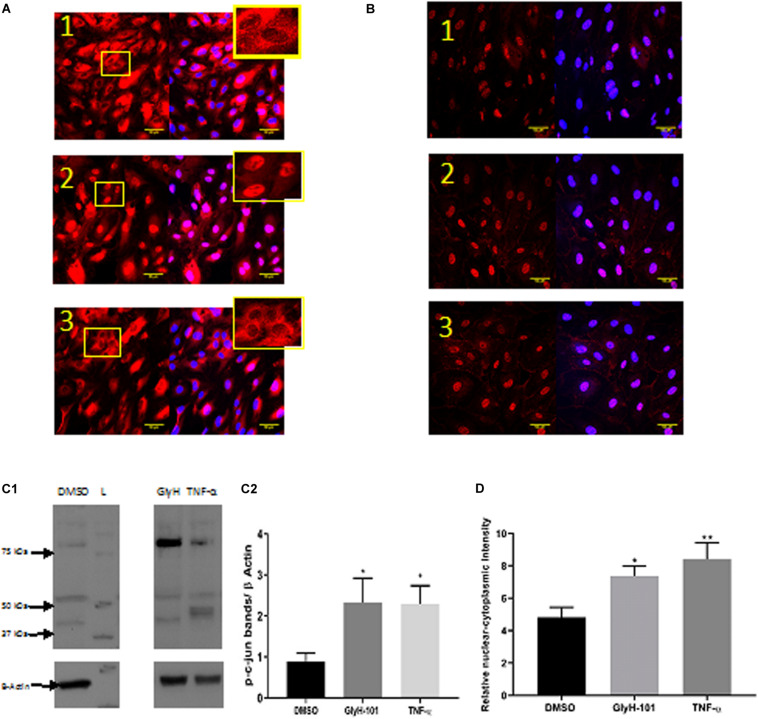
The effect of GlyH-101 on cell signaling. **(A)** NF-κB immunocytochemistry of HLMVECS after 1 h of 1; DMSO (0.1%), 2; TNF-α (10 ng/ml), 3; GlyH-101 (20 μM). No primary antibody controls showed no positive staining. **(B)** p-c-jun immunocytochemistry of HLMVECs after 30 min of 1; DMSO (0.1%), 2; TNF-α (10 ng/ml), 3; GlyH-101 (20 μM). No primary antibody controls showed no positive staining. **(C)** p-c-jun western blotting. **(C1)** image of whole cell lysate after treating HLMVECs with DMSO (0.1%), TNF-α (10 ng/ml), GlyH-101 (20 μM) for 30 min. L; molecular weight ladder. **(C2)** quantification p-c-jun on western blots from three independent experiments. Data were analyzed with one-way ANOVA and Fisher *post hoc* test. (* *p* < 0.05 compared with DMSO control, *n* = 3). **(D)** Immunocyto- chemistry quantification of relative nuclear to cytoplasmic p-c-jun intensity, data represent three independent experiments, in which 20 cells were quantified from each condition. Data were analyzed with one-way ANOVA, and Fisher test (* *p* < 0.05, ** *p* < 0.01,compared with DMSO control, *n* = 3).

Functional CFTR was reported to be a negative regulator of NF-κB and inflammatory cell signaling (Vij et al., 2009). We therefore investigated the effect of CFTR inhibition on activation of NF-kB and AP-1, two key ROS-dependent signaling pathways leading to IL-8 expression (Hoffmann et al., 2002), with TNF-α (10 ng/ml) as a positive control stimulus. While TNF-α clearly stimulated nuclear translocation of the NF-kB p65 subunit (Figure 10A, panel 2), CFTR inhibition with GlyH-101 (20 μM) had no effect (Figure 10A, panel 3). However, both GlyH-101 and TNF-α significantly (*p* = 0.0326 with Cohen’s *d* = 1.86 and *p* = 0.035 with Cohen’s *d* = 2.37, respectively) increased phosphorylation of the AP-1 c-Jun subunit (Figures 10C1,C2) and significantly (*p* = 0.0476 with Cohen’s *d* = 1.96, and *p* = 0.0094 with Cohen’s *d* = 1.99, respectively) increased its nuclear translocation (Figures 10B,D).

## Discussion

### Summary of Key Findings

We have shown that lung microvascular endothelial cells respond to CFTR inhibition with significantly increased levels of ROS, increased IL-8, VEGF and SOD-2 expression, and a significant 70% decrease in Nrf2 protein levels. GlyH-101, a CFTR-specific inhibitor, increased IL-8 expression via ROS-dependent activation of AP-1 signaling with increased nuclear phospho-c-Jun in HLMVECs, but no evidence of NF-κB activation. Further, CFTR inhibition enhanced TNFα-stimulated IL-8 expression, which occurs via both AP-1 and NF-κB signaling pathways. We show that dysfunctional CFTR enhanced EGFR-dependent expression of IL-8, which was inhibited using the EGFR tyrosine kinase specific inhibitor AG1478. Conversely, the increased expression of VEGF following CFTR inhibition was significantly enhanced in the presence of AG1478, and in this respect these endothelial cells differ from airway epithelial cells. Overall, the data indicate that suppression of endothelial CFTR activity activates production of intracellular ROS, and ROS-dependent signaling cascades leading to activation of nuclear transcription factors involved in IL-8 and VEGF synthesis.

### CFTR Expression

Endothelial CFTR expression was confirmed in the present study, but appeared to be lower in our model than previously reported in HLMVECs (Tousson et al., 1998), possibly reflecting growth on collagen IV, which is an exclusive component of basement membranes that regulates endothelial cell function (Wang and Su, 2011). Levels of expression varied greatly between HLMVEC preparations, as reported for other tissues (Riordan et al., 1989), and appeared to be largely associated with intracellular organelles with punctate staining indicating localization in the endoplasmic reticulum surrounding the nucleus, as previously reported in endothelial cells (Tousson et al., 1998).

Since we found that the CFTR inhibitor GlyH-101 does not activate endothelial NF-κB (Figure 10), the increased expression of CFTR following CFTR inhibition is unlikely to be via activation of the TNF-R adaptor molecule, TRADD, and NF-κB activation, as has been previously described in bronchial epithelial cells (Wang et al., 2016). However, Nrf2 has an inhibitory effect on CFTR expression (Rene et al., 2010) and repression of CFTR expression occurs under conditions of prolonged oxidative stress in bronchial epithelial cells (Zhang et al., 2015). Thus, the significantly reduced levels of endothelial Nrf2 we detected in HLMVECs following CFTR inhibition (Figure 5) may contribute to the observed increase in endothelial CFTR expression following treatment with GlyH-101.

However, the low levels of mature CFTR in the plasma membrane were activated by the cAMP-elevating agents, forskolin plus IBMX, to cause cell depolarization. This response was inhibited by GlyH-101, a water-soluble glycine hydrazide reported to bind to CFTR externally in the channel pore where, at 10 μM, it rapidly and completely inhibited CFTR chloride channel activity in human airway epithelial cells (Muanprasat et al., 2004). The cAMP-elevating agents stimulated membrane depolarization in both HLMVECs and 16HBEs and the non-CFTR expressing HEK293 cells. Significant inhibition of the response by GlyH-101 in HLMVECs and 16HBEs indicated that depolarization was partly due to CFTR. However, other cAMP-activated channels, such as cAMP-gated non-selective cation channels (Nilius and Droogmans, 2001), which are not inhibited by GlyH-101, may also contribute to membrane depolarization in these cells.

### CFTR and Oxidative Stress

Oxidative stress is a key contributor to vascular endothelial dysfunction in people with CF (Tucker et al., 2019). In the present study, increased intracellular oxidative stress in HLMVECs and 16HBEs was demonstrated in response to CFTR inhibition. Chen et al. (2008) previously reported an increase in intracellular H_2_O_2_ following 72 h of CFTR inhibition in human bronchial epithelial cells. The difference lies in the rapid response in HLMVECs, in which intracellular ROS were significantly induced over 5–30 min following CFTR inhibition with GlyH-101 and was reflected in a significant increase in intracellular H_2_O_2_ concentrations at 30 min compared to baseline levels. H_2_O_2_ is a highly diffusible cell signaling molecule with multiple redox-sensitive molecular targets. These include oxidative inactivation of protein phosphatases and direct or indirect activation of kinases, converging in the regulation of transcription factor activity and effects on endothelial cell function via increased expression of growth factors including VEGF and transactivation of growth factor receptors including EGFR (Bretón-Romero and Lamas, 2014; Kim and Byzova, 2014; Marinho et al., 2014).

The ROS signal following CFTR inhibition was completely ablated in the presence of NAC, despite the fact that NAC has only weak direct antioxidant properties toward superoxide anions and H_2_O_2_ (Aldini et al., 2018). However, as a precursor of cysteine and thus of glutathione (GSH) synthesis, NAC is effectively an indirect antioxidant, GSH being a direct antioxidant and a substrate of several antioxidant enzymes (Aldini et al., 2018). CFTR may be a major transporter of GSH in both epithelial and endothelial cells (Roum et al., 1993; Gao et al., 1999). Impaired CFTR activity diminishes both intracellular and extracellular GSH levels in bronchial epithelial cells (de Bari et al., 2018), and impaired endothelial CFTR activity may contribute to the systemic deficiency in GSH in CF (Roum et al., 1993). A rapid, within 10 min, significant 50% decrease in GSH in airway epithelial cells following CFTR inhibition with the CFTR inhibitor CFTRinh-172 was recently reported (de Bari et al., 2018). Thus, if CFTR inhibition similarly lowers intracellular GSH concentrations in HLMVECs, this may contribute to the observed rapid increase in ROS/H_2_O_2_ following treatment with GlyH-101. On the other hand, the significant decrease in ROS observed in the presence of 10 mM NAC may reflect an increase in intracellular GSH, as previously reported for human pulmonary vascular endothelial cells treated with 10 mM NAC (Hashimoto et al., 2001).

Major sources of ROS production in vascular endothelial cells include the mitochondria, endoplasmic reticulum, plasma membrane and cytosolic enzymes (Bretón-Romero and Lamas, 2014). Mitochondrial defects and increased production of ROS, although controversial (Schwarzer et al., 2007), have been reported in CF lung (Valdivieso and Santa-Coloma, 2013; Atlante et al., 2016), and gut (Kleme et al., 2018) epithelial cells. However, the mitochondrially targeting antioxidant MitoQ did not attenuate ROS production in HLMVEC or 16HBEs, and since the NOX/Duox inhibitor, diphenyleneiodonium (DPI) at 10 μM, failed to inhibit H_2_O_2_ formation, another source of endothelial ROS is indicated (Cai, 2005), which warrants further investigation.

Cystic fibrosis transmembrane conductance regulator dysfunction in airway epithelial cells was previously reported to significantly increase SOD-2, while reducing catalase, expression (Chen et al., 2008). Similarly, inhibition of endothelial CFTR induced a significant increase in SOD-2, albeit in the absence of a significant decrease in catalase expression.

SOD-2 is localized in the mitochondrial matrix and catalyzes the dismutation of O2^⋅–^ to H_2_O_2_. The observed increase in SOD-2 expression 16–24 h following CFTR inhibition, in the absence of any increase in catalase expression, would serve to maintain relatively high intracellular H_2_O_2_ levels in our endothelial cell cultures, as previously reported in airway epithelial cells over 72 h (Chen et al., 2008). SOD-2 expression is not reported to be regulated by Nrf2 (Türei et al., 2013) and, considering the loss of Nrf2 function over 16–24 h (Figure 5), we propose that SOD-2 expression is upregulated in response to H_2_O_2_ -mediated activation of other redox sensitive transcription factors (Yoshioka et al., 1994).

### CFTR and Nrf2 Expression

Previous work has demonstrated, in intestinal epithelial cells, that CFTR knockout significantly increased mitochondrial levels of H_2_O_2_ and decreased mitochondrial levels of Nrf2 (Kleme et al., 2018). As in bronchial epithelial cells (Chen et al., 2008), CFTR inhibition in HLMVECs led to a 70% decrease in endothelial Nrf2 expression in the present study, although further work is needed to demonstrate the subcellular organelle distribution of Nrf2 in HLMVECs.

Since Nrf2 is a transcription factor and a master regulator of the adaptive cellular response to oxidative stress in endothelial cells, one would expect that oxidative stress would induce an adaptive response (reviewed in 53). Adaptive responses are characterized by increased Nrf2 expression and activity, upregulated antioxidant response element (ARE)-regulated gene expression with co-ordinated induction of endogenous cytoprotective enzymes. In view of our evidence of oxidative stress in endothelial cells following CFTR inhibition (increased ROS/H_2_O_2_), the observed decrease in Nrf2 is a paradox, but one which was also previously reported in airway epithelial cells (Chen et al., 2008). H_2_O_2_ is further metabolized by catalase in the peroxisomes or by the glutathione peroxidase, peroxiredoxin/thioredoxin system, isoforms of which are found in the mitochondria and the cytosol, and whose expression is normally regulated by Nrf2. However, expression of these cytoprotective proteins is decreased in CF epithelial cells, and CF nasal and lung tissue, and this was linked to the decreased expression of Nrf2 (Chen et al., 2008).

Nuclear related factor 2 is normally maintained at low levels intracellularly by its association with Kelch-like ECH-associated protein 1 (Keap-1), a substrate adapter in an E3 ubiquitin ligase complex, leading to Nrf2 ubiquitination and proteosomal degradation (Rada et al., 2011; Marinho et al., 2014). H_2_O_2_ oxidizes critical cysteine residues in Keap-1, inducing conformational changes affecting the interaction between Keap-1 and Nrf2, thereby inhibiting Nrf2 ubiquitination and degradation. In this way, stabilization of Nrf2 by H_2_O_2_ leads to translocation and accumulation of Nrf2 in the nucleus, upregulation of ARE-driven gene expression and coordinated induction of endogenous cytoprotective proteins. However, Nrf2 can also be tagged for degradation by phosphorylation mediated by glycogen synthase kinase-3β (GSK-3β). Phosphorylated Nrf2 is recognized and bound by the β-transducin repeat containing protein (β-TrCP), a substrate adapter forming an E3 ligase complex, followed by ubiquitination and proteosomal degradation of Nrf2. Since GSK-3β is *inhibited* by PKB/Akt-mediated phosphorylation, and H_2_O_2_ is an activator of the PI3K/Akt pathway, then H_2_O_2_ can also activate Nrf2 in a Keap-1 independent manner (Rada et al., 2011; Marinho et al., 2014).

**FIGURE 11:**
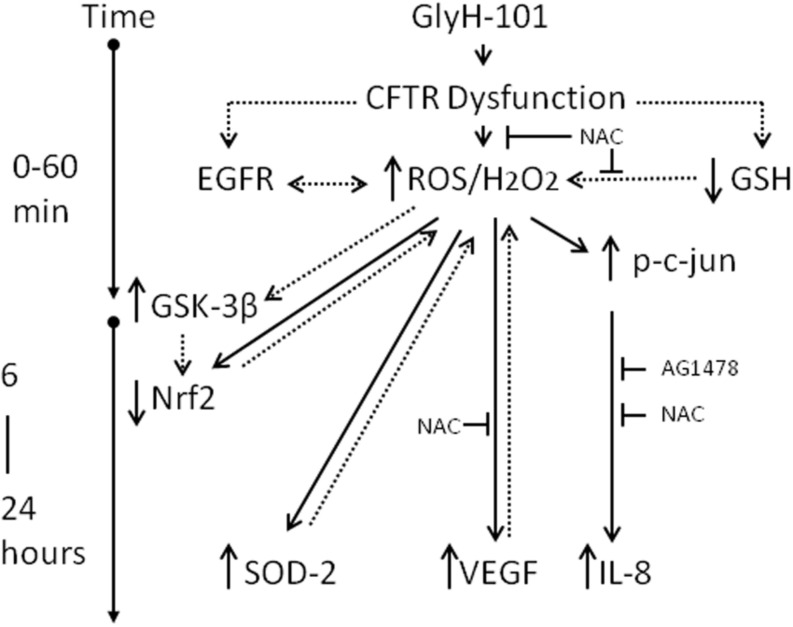
Schematic of the impact of CFTR dysfunction in HLMVEC on oxidative stress, inflammation and angiogenesis. Specific pharmacological inhibition of endothelial CFTR activity is associated with an increase in intracellular ROS (5 min) and hydrogen peroxide (30 min), an increase in phosphorylation of c-jun and activation of AP-1 (30 min) leading to transcription of IL-8 (16–24 h). These responses are inhibited by the antioxidant NAC and the EGFR inhibitor AG1478, indicating a role for EGFR-mediated ROS/H_2_O_2_ generation in the induction of IL-8 synthesis. The increase in ROS/H_2_O_2_ is associated with increased VEGF and SOD2 expression which serve to sustain high levels of intracellular hydrogen peroxide, via effects on VEGFR and superoxide dismutation, respectively. Under these conditions Nrf2, the master regulator of oxidative stress, is significantly depleted, which is proposed to occur via prolonged exposure to high levels of H_2_O_2_, activation of GSK-3β and labeling of Nrf2 for β-TrCP recognition, ubiquitination and degradation. The suppression of Nrf2 levels to below normal will further sensitize endothelial cells to oxidative stress by reducing expression of antioxidant genes. Dotted lines indicate proposed pathways. T indicates inhibition of a pathway.

However, while short-term exposure to H_2_O_2_ inhibits GSK-3β and activates Nrf2, prolonged exposure to high H_2_O_2_ concentrations *activates* GSK-3β, which could terminate the Nrf2 signal by degradation of Nrf2 through the β-TrCP pathway leading to lower than normal levels of Nrf2 (Rada et al., 2011; Marinho et al., 2014). These pathways are outlined in Figure 11. Thus it would be of future interest to investigate if antioxidants, such as NAC, could prevent the loss of Nrf2 following CFTR inhibition in endothelial cell cultures.

### Nrf2 as a Therapeutic Target

Since, Nrf2 expression is central to limiting oxidative stress and inflammation, the reduced expression of Nrf2 in CF airway epithelium (Chen et al., 2008), and now also demonstrated in pulmonary endothelial cells with dysfunctional CFTR, strongly indicate Nrf2 may be a therapeutic target to limit oxidative stress and airway inflammation by increasing its expression and activation in CF. Such an approach has been demonstrated using the synthetic triterpenoid CDDO (2-cyano-3,12-dioxooleana-1,9 (Peters et al., 2015)-dien-28-oic acid) in airway epithelial cell and animal models of CF (Nichols et al., 2009). Further, since CDDO activates PI3K-PKB/Akt signaling (reviewed in 53), the increase in Nrf2 activity in CF airway epithelial cells following treatment with CDDO (Nichols et al., 2009), suggests the reduced Nrf2 activity in preclinical models of CF lung disease is associated with GSK-3β activity. It would therefore be of future interest to investigate the effect of specific pharmacological activators of PI3K-PKB/Akt and inhibitors of GSK-3β activity on Nrf2 activity in pulmonary endothelial cells. Other Nrf2 activators include natural products such as curcumin (Balogun et al., 2003) and sulforaphane, an isothiocyanate enriched in broccoli sprouts, which has been proposed as a dietary supplement to activate Nrf2 (Galli et al., 2012).

In addition to regulation by proteosomal degradation, cytosolic Nrf2 levels are regulated at the stage of Nrf2 gene transcription. Amongst factors that increase Nrf2 transcripts is Nrf2 itself (Suzuki et al., 2013), possibly contributing to the overall significant decrease in Nrf2 levels following CFTR inhibition. Of interest, is the recent observation that clinically approved CFTR modulators rescue Nrf2 dysfunction in CF airway epithelial cells (Borcherding et al., 2019). The effect of CFTR modulators on endothelial Nrf-2 expression and activity remains to be investigated.

### CFTR and VEGF Expression

Increased VEGF expression and peribronchial angiogenesis is a feature of the CF airway. VEGF promotes angiogenesis and increased vascular permeability, enhancing inflammatory cell recruitment and plasma exudation in the airway. Inhibition of airway epithelial CFTR increased VEGF expression, an effect that was dependent on EGFR activity and inhibited by the receptor tyrosine kinase inhibitor AG1478 (Martin et al., 2013). Further, CF airway epithelial cells have increased EGFR activity and phosphorylation compared to normal cells (Stolarczyk et al., 2018). Thus it was suggested that EGFR tyrosine kinase inhibitors, such as AG1478, might be useful in the treatment of CF. However, the findings in the present study clearly indicate that such an approach further enhances the increased VEGF expression observed following CFTR inhibition in HLMVECs, and in this respect these endothelial cells differ from epithelial cells.

Conversely, EGFR inhibition reduced IL-8 expression in CFTR-inhibited epithelial cells (Kim et al., 2013) and, as demonstrated in the present study, also in HLMVECs. Since anti-inflammatory therapies targeting inhibition of EGFR are likely to increase VEGF expression in endothelial cells, other approaches such as those that target oxidative stress (Kim and Byzova, 2014) are needed. This notion is supported by our evidence that the antioxidant NAC significantly reduced ROS, and both IL-8 and VEGF expression in HLMVECs. In addition, others have recently demonstrated that ingestion of an antioxidant cocktail can improve vascular endothelial cell function and oxidative stress in patients with CF (Tucker et al., 2019).

### CFTR and IL-8 Expression

Dysfunctional CFTR significantly increased endothelial IL-8 expression both in the absence and presence of TNFα, a pro-inflammatory cytokine that enhances neutrophil responses in CF airways (Nichols and Chmiel, 2015). Increased expression of IL-8 in pulmonary epithelial cells with dysfunctional CFTR was previously associated with increased activation of the transcription factor NF−κB (Vij et al., 2009), which works with other transcription factors, including AP−1, as a central transcriptional regulator of airway inflammation in CF (Nichols and Chmiel, 2015). Diminished Nrf2 levels are proposed to exacerbate activation of redox sensitive pathways including activation of AP-1 and NF-κB signaling leading to increased IL-8 expression (Hoffmann et al., 2002) and activation of EGFR signaling pathways (Burgel and Nadel, 2008). GlyH-101 induced a rapid (within 30 min) significant increase in the cellular abundance of phospho-c-Jun, including an increase in the concentration of nuclear phospho-c-Jun. These changes are indicative of increased c-Jun expression and activation of AP-1 signaling, which contribute to a self-amplifying cycle of increased c-Jun abundance (Marinho et al., 2014). However, no nuclear translocation of p65, an indicator of NF-κB activation, was seen following treatment of HLMVECs with GlyH-101 alone for up to 1 h, ruling out a role for NF-κB activation via the canonical pathway in the IL-8 response to inhibition of CFTR activity. Similarly, the CFTR inhibitor CFTRinh172 also did not induce NF-κB activation and nuclear translocation in airway epithelial cells (Perez et al., 2007).

Unlike GlyH-101, TNFα induced activation of both NF-κB and AP-1 signaling, leading to significantly increased IL-8 expression in HLMVECs, beyond the response to GlyH-101 alone. Of particular interest in this respect is the recent report that JNK is activated in CF epithelial cells and further activated in response to TNFα (Saadane et al., 2011).

The complete inhibitory effect of AG1478 on the GlyH101-induced increase in IL8 synthesis indicates that this response occurred via a signaling cascade leading to ligand-dependent EGFR signaling, as previously described for human airway epithelial cells exposed to the CFTRinh172 (Kim et al., 2013). EGFR activation leads to rapid (within 5 min) generation of  H_2_O_2_ (Bae et al., 1997) by mechanisms which are not yet described (Weng et al., 2018), with immediate response gene expression, including JUN and FOS, occurring over the first 45 min (Avraham and Yarden, 2011). Our data therefore indicate the early, 30 min, increase in phospho-c-Jun observed in response to GlyH-101 is mediated via H_2_O_2_-activated EGFR signaling (Weng et al., 2018) and involved in AP-1-mediated IL-8 expression.

The inhibitory effect of NAC on IL-8 production in response to GlyH-101 in the presence of TNFα further indicates that ROS are involved in signaling pathways leading to IL-8 synthesis in endothelial cells. Following CFTR inhibition, GlyH-101 appears to act solely through the EGFR cascade and AP-1 activation, while TNF-α activates both NF-κB and AP-1 signaling pathways. ROS contribute to EGFR activation, and activation of NF-κB and AP-1 signaling pathways, as described above. Whether endothelial oxidative stress further contributes to increased IL-8 gene expression through remodeling of chromatin structure and increased histone H4 acetylation at the IL-8 promoter, as seen in CF airway epithelial cell models (Bartling and Drumm, 2009), is not known.

### GlyH-101 Is a Specific Inhibitor of Endothelial CFTR

Some degree of non-specificity for GlyH-101 has been reported in murine epithelial cell lines (Melis et al., 2014), although these effects appear to be species specific (Stahl et al., 2012). In murine cells, GlyH-101 inhibited the volume-sensitive outwardly rectifying Cl^–^ conductance (VSORC) and the calcium dependent Cl^–^ conductance (CaCC), which in endothelial cells is predominantly TMEM16A. However, although TMEM16A activity was reported to negatively regulate pro-inflammatory cytokine, including IL-8, synthesis in human CF bronchial epithelia (Veit et al., 2012), TMEM16A is a positive regulator of endothelial ROS (Ma et al., 2017), and its inhibition would lead to decreased ROS in endothelial cells, and not the increase reported in the current study, supporting the involvement of CFTR in the observed responses to GlyH-101. In our control experiments, specific inhibition of other chloride ion channels, VRAC and TMEM16A had no effect on IL-8 expression, and GlyH-101 had no effect on IL-8 expression in HEK-293 cells that do not express CFTR. Together these experiments indicate that the observed effects of GlyH-101 in HLMVECs reflect inhibition of CFTR activity without off-target effects.

Others reported non-specific effects of GlyH-101 on mitochondrial respiration and a rapid increase in ROS levels that were independent of CFTR channel inhibition, although these were largely abrogated in the presence of 10% serum, and under these conditions GlyH-101 had no effect on basal oxygen consumption (Kelly et al., 2010). Serum (5%) was present in our HLMVEC cultures to provide a reducing extracellular environment (Chan et al., 2001) and as a source of albumin to increase intracellular glutathione that protects pulmonary cells from oxidant-mediated cytotoxicity (Cantin et al., 2000). Under these conditions, but not in the absence of serum, NAC acts as an anti-oxidant (Chan et al., 2001), as we observed. Further, we saw no effect of MitoQ on the GlyH-101 stimulated ROS production in HLMVECs or 16HBEs, ruling out a direct effect of GlyH-101 on mitochondrial ROS.

### Therapeutic Relevance

Systemic inflammation and oxidative stress are characteristics of people with CF and it is proposed that dysfunctional endothelial cells contribute to these changes in the circulation in people with CF (Declercq et al., 2019; Causer et al., 2020). Further, systemic inflammation, oxidative stress and endothelial dysfunction were proposed to be major risk factors for cardiovascular disease in the aging CF population (Reverri et al., 2014). Our data now point to a role for dysfunctional endothelial CFTR in systemic oxidative stress and inflammation in CF.

However, to date, there are limited options for anti-inflammatory therapy for CF (Cantin et al., 2015). Targeting endothelial CFTR for correction and/or potentiation would seem to be a logical therapeutic approach, as small molecule correctors and potentiators are already in development to restore epithelial CFTR function. It was surprising that 6 months treatment with ivacaftor in CF patients with the G551D mutation improved lung function, and reduced sweat chloride, indicating an effect on CFTR function, without an effect on pulmonary inflammation (Rowe et al., 2014). Systemic inflammation was not measured in the study, so it is not possible to appreciate the effect of the oral drug, ivacaftor, on endothelial function. The combination of lumacaftor with ivacaftor (Orkambi) for delF508 patients was approved in 2015, but to our knowledge no studies have reported effects on systemic inflammation.

Correction of CFTR expression in the bronchial epithelium by gene transfer in 10–25% of cells in a population was sufficient to restore normal chloride conductance and epithelial function (Johnson et al., 1992; Zhang et al., 2009). However, it is currently unclear whether low-level expression in many cells (e.g., 10% of residual CFTR expression) or a low number of cells expressing high levels of CFTR are required to restore normal function and achieve clinical benefit after gene therapy (Griesenbach et al., 2015). Recent revised estimates of the number of endothelial cells in the human body indicate a total of 6 × 10^11^ cells (Sender et al., 2016), and low level of expression of CFTR in all of them may be sufficient to maintain normal barrier, anti-inflammatory and anti-oxidant endothelial function.

In addition to inherited defects in CFTR in people with CF, inhibition of CFTR on endothelial cells increased the loss of barrier function induced by exposure of endothelial cells to cigarette smoke (Brown et al., 2014). Raju et al. (2013) showed that smoking causes systemic CFTR dysfunction and that acrolein present in cigarette smoke mediates CFTR defects in extrapulmonary tissues in smokers. Endothelial dysfunction is associated with loss of lung function, severity of disease and reduced exercise capacity in COPD (Green and Turner, 2017). Together, these findings indicate that acquired loss of endothelial CFTR function in response to cigarette smoking may be relevant to the development of vascular disease and other co-morbidities in COPD, and a further target for therapy.

### Limitations

While our data identify CFTR expressed in human lung microvascular endothelium as a controller of oxidative stress, ROS-mediated cell signaling and inflammatory responses, it was based on a limited number (*n* = 3–6) of independent experiments. In addition, this study was performed in a model in which normal endothelial CFTR function was inhibited pharmacologically. To better understand the role of dysfunctional endothelial CFTR in people with CF, studies could be conducted using primary endothelial cells or cell lines from people with CF in cell culture models under shear flow, for example, and eventually in the *in vivo* situation, given the importance of various cellular interactions in inflammatory responses.

## Conclusion

In conclusion, our study points to restoring endothelial CFTR and Nrf2 activity as therapeutic targets in people with CF, and supports the use of systemic antioxidants to reduce vascular inflammation and angiogenesis in CF.

## Data Availability Statement

All datasets generated for this study are included in the article/supplementary material.

## Author Contributions

MK, TS-W, AC, and JS acquired and statistically analyzed the data. ZS, AS, DG, AL, DL, and JS contributed to conception and design of the study. JS wrote the first draft of the manuscript. All authors contributed to manuscript revision and approval of the submitted version.

## Conflict of Interest

The authors declare that the research was conducted in the absence of any commercial or financial relationships that could be construed as a potential conflict of interest.
